# HTLV-1 in Ophthalmology

**DOI:** 10.3389/fmicb.2020.00388

**Published:** 2020-03-11

**Authors:** Koju Kamoi

**Affiliations:** ^1^Department of Ophthalmology and Visual Science, Graduate School of Medical and Dental Sciences, Tokyo Medical and Dental University, Tokyo, Japan; ^2^HTLV-1 Uveitis/ATL-Related Ocular Disease Clinic, Research Hospital, The Institute of Medical Science, The University of Tokyo, Tokyo, Japan; ^3^Department of Hematology/Oncology, Research Hospital, The Institute of Medical Science, The University of Tokyo, Tokyo, Japan

**Keywords:** human T-cell leukemia virus type 1, HTLV-1 uveitis, ATL-related ocular diseases, ocular inflammation, uveitis, ocular infiltration

## Abstract

Human T-cell leukemia virus type 1 (HTLV-1) was the first retrovirus described as a causative agent for human disease. In the field of ophthalmology, a close relationship between HTLV-1 infection and uveitis was identified through a series of clinical and laboratory studies in the late 1980s–1990s. Since then, HTLV-1-related ocular manifestations such as keratoconjunctivitis sicca, interstitial keratitis, optic neuritis and adult T-cell leukemia/lymphoma (ATL)-related ocular manifestations have continuously been reported. During the three decades since the association between HTLV-1 and ocular pathologies was discovered, ophthalmic practice and research have advanced with the incorporation of new technologies into the field of ophthalmology. Accordingly, new findings from recent research have provided many insights into HTLV-1-associated ocular diseases. Advanced molecular technologies such as multiplex polymerase chain reaction (PCR)/broad-range PCR using ocular samples have enabled rapid and accurate diagnosis. Advanced ophthalmic technologies such as widefield fundus camera and optical coherence tomography (OCT) have clarified various features of HTLV-1-associated ocular manifestations, and identified characteristics such as the “knob-like ATL cell multiple ocular infiltration” (KAMOI) sign. Advanced drug delivery methods such as intravitreal injection and sub-Tenon injection have led to progress in preventing disease progression. This article describes global topics and the latest research findings for HTLV-1-associated ocular diseases, with reference to a large-scale nationwide survey of ophthalmologists. Current approaches and unmet needs for HTLV-1 infection in ophthalmology are also discussed.

## Introduction

“Seeing is believing” is an old but profound proverb. In fact, we humans typically obtain 80% of our information on the outside world through sight. Visual impairment thus exerts a major impact on quality of life. To see things clearly, smooth light penetration throughout the eye is essential. Visual recognition can be obtained when light from the outside world passes through the cornea, anterior chamber, lens, and vitreous to reach the retina. Thereafter, photonic stimuli are converted into neural signals that reach the brain through the optic nerve. Disturbance of even a single part of this transmission pathway from the eye results in visual impairment.

Human T-cell leukemia virus type 1 (HTLV-1) is a retrovirus that has been attracting worldwide attention since it was found that fully half of Aboriginal adults in central Australia are infected by the virus (Gruber, 2018). Looking around the world, an estimated 20 million people are infected by this virus. HTLV-1 was discovered in the 1980s (Coffin, 2015) and was found to cause adult T-cell leukemia/lymphoma (ATL), HTLV-1-associated myelopathy (HAM), and HTLV-1-associated ocular diseases (Watanabe, 1997). HTLV-1-associated ocular diseases are mainly categorized into HTLV-1 uveitis (HU) and ATL-related ocular manifestations. Even now, HU is the most frequent ocular inflammatory disease in endemic areas (Terada et al., 2017a), which is a concerning issue. Ophthalmologists and researchers have been tackling this virus and gaining knowledge regarding HTLV-1-associated ocular diseases over the past three decades.

In this article, I discuss: (1) the history of HTLV-1-associated ocular diseases; (2) the global distribution of HTLV-1-related eye diseases; (3) recent advances in ophthalmology and their application to HTLV-1-associated ocular diseases; (4) current knowledge regarding HTLV-1-associated ocular diseases; (5) current concerns; and (6) unmet research needs. I believe this article will prove useful for organizing disparate information on HTLV-1-associated ocular diseases.

## History of HTLV-1-Associated Ocular Diseases

To date, many ophthalmologists have reported a variety of ocular manifestations related to HTLV-1 infection. From the latter half of the 1980s to the early 1990s, the relationship between HTLV-1 and ocular inflammation, i.e., uveitis, has been reported from the perspective of clinical research among HTLV-1 carriers (Ohba et al., 1989; Nakao et al., 1991; Mochizuki et al., 1992c; Nakao and Ohba, 1993). Confirmation of a relationship between HTLV-1 and uveitis was achieved in the 1990s through a series of laboratory studies, which identified a direct association between HTLV-1 and uveitis, i.e., HU (Kamoi and Mochizuki, 2012b).

As for other ocular manifestations among HTLV-1 carriers, a relationship between HTLV-1 and Sjögren’s syndrome/keratoconjunctivitis sicca (KCS) was reported in the early 1990s (Eguchi et al., 1992; Terada et al., 1994). Interstitial keratitis (IK) and other corneal lesions such as corneal haze and central opacities with thinning, bilateral immunoprotein keratopathy, peripheral corneal thinning, scarring, and neovascularization were reported in the early 2000s (Buggage et al., 2001; Merle et al., 2001).

HTLV-1 is known to cause HAM, a progressive neurologic disease. HU was reported to accompany HAM in the late 1980s (Ohba et al., 1989). Sjögren’s syndrome/KCS was also described in patients with HAM in the late 1990s–2000s (Merle et al., 1996; Yamamoto et al., 1999, 2002; Patronas et al., 2006; Pinheiro et al., 2006). Optic neuritis accompanied by HAM was also reported in the late 1990s (Yoshida et al., 1998).

ATL is one of the most aggressive leukemias, and has been identified to be caused by HTLV-1 infection. Ocular manifestations among ATL patients started to be reported around the late 1980s–1990s (Ohba et al., 1989; Goto et al., 1993). However, all manifestations have been reported as case reports or case series because of the rarity of cases and the difficulty of documentation resulting from the severe condition and high fatality rate of patients. In the late 2010s, we performed a nationwide survey and identified the frequency of ATL-related ocular manifestations (Kamoi et al., 2018).

## Global Distribution of HTLV-1-Associated Ocular Diseases

HTLV-1-associated ocular disease is estimated to occur all over the world, corresponding to the geographical distribution of HTLV-1 infection (Gessain and Cassar, 2012). Here, the various types of reports published from across the world were analyzed, classified and summarized from the perspective of relationships between HTLV-1-associated ocular diseases and region/ethnic susceptibility.

### HTLV-1 Carriers and Ocular Disease

The first evidence of links between HTLV-1 and ocular diseases was reported from Japan. The most frequently reported HTLV-1-associated ocular disease is HU (Kamoi and Mochizuki, 2012b). Ophthalmologists have reported on HU from Hokkaido to Kyushu, with cases described from all over Japan (Mochizuki et al., 1992b; Goto et al., 1994; Kawamoto and Hayasaka, 1994; Ohba et al., 1994a, b; Ishioka et al., 1995; Takahashi et al., 2000; Miyanaga et al., 2009; Kase et al., 2013; Terada et al., 2017a). As for other areas of the world, clinical research into HU has been reported from the United States (Beilke, 1995), Martinique (Merle et al., 1996), Britain (Sandy et al., 1996), and Brazil (Yamamoto et al., 1999). Most recently, HU was also reported from Central Australia (Chew et al., 2018). HU is thus distributed all around the globe.

As for other ocular manifestations, the relationship between Sjögren’s syndrome/KCS and HTLV-1 infection was first noted in Japan (Eguchi et al., 1992). Corneal manifestations have since also been published from all over the world, such as Martinique (Merle et al., 1996), Brazil (Yamamoto et al., 2002; Pinheiro et al., 2006; Rathsam-Pinheiro et al., 2019), and the United States (Buggage et al., 2001).

### HAM and Ocular Disease

HU has been reported to accompany HAM in Japan (Ohba et al., 1989; Terada et al., 2017a; Nakao et al., 2018). Sjögren’s syndrome/KCS has also been described in patients with HAM in Japan, Martinique (Merle et al., 1996), and Brazil (Yamamoto et al., 1999, 2002). A report from Brazil showed that HAM patients displayed more ophthalmologic symptoms than asymptomatic HTLV-1-infected individuals, with significantly higher frequencies of KCS and immunologic alterations (Pinheiro et al., 2006). A report from the United States noted corneal opacity in HAM patients with hypergammaglobulinemia (Patronas et al., 2006). Other research from Japan has indicated that optic neuritis was accompanied by HAM (Yoshida et al., 1998).

### ATL and Ocular Disease

ATL-related ocular diseases are rare (Kamoi and Mochizuki, 2012a), resulting in sporadic case reports. Analyzing such reports, ocular infiltration of ATL was the most frequent manifestation, observed in almost all tissues in and around the eye, and with reports mainly arising from Japan (Goto et al., 1993; Shibata et al., 1997; Mori et al., 2003; Yoshikawa et al., 2005; Miyamura et al., 2009; Liu et al., 2010; Maruyama et al., 2013; Kamoi et al., 2016; Hirano et al., 2017; Shimono et al., 2017; Sabundayo et al., 2018; Yamada et al., 2019), followed by Martinique (Merle et al., 2005, 2016; Fares et al., 2018) and the United States (Liu et al., 2010; Larson et al., 2012). In 2018, our nationwide survey in Japan confirmed that the most frequent ATL-related ocular manifestation was ATL infiltration (Kamoi et al., 2018).

### Race and Susceptibility of Ocular Diseases

Scrutinizing these reports from an ethnic perspective, most HTLV-1 ocular manifestations have been identified in Japanese and Caribbean populations. These ethnic groups are known to show high rates of HU, HU accompanied by HAM, corneal lesions, and ATL-related ocular manifestations. Ophthalmic researchers in Brazil indicated that Caucasian and mixed-ethnicity groups also experience HU and KCS (Yamamoto et al., 1999). African (Beilke, 1995), and Hispanic (Larson et al., 2012; Rathsam-Pinheiro et al., 2019) and Australian Aboriginal groups (Chew et al., 2018) also show HU. Analysis of ophthalmic research from around the world suggests that susceptibility to HTLV-1-associated ocular diseases does not appear to differ among ethnic groups.

## Recent Advances in Ophthalmology and Applications to Htlv-1-Associated Ocular Diseases

### Advances in Imaging Technology

The traditional fundus camera can capture around 30% of the retinal field with full-field flash illumination. A widefield fundus camera, the Optos© (Optos PLC, Dunfermline, United Kingdom), was developed in Scotland and became commercially available in the 2000s. The widefield fundus camera can capture more than 80% of the fundus in a non-invasive manner and without requiring mydriasis. The advantage of the widefield fundus camera is visualizing a retinal peripheral lesion in one picture, as this is not possible with a traditional fundus camera. Such cameras are now an essential tool among ophthalmologists for the identification of peripheral retinal and vascular pathologies. The concept underlying widefield imaging has been applied to other fundus-visualizing technologies, such as widefield fluorescein angiography for retinal vessel analysis, widefield indocyanine green angiography for choroidal vessel analysis, and widefield autofluorescence for retinal pigment epithelium analysis.

**FIGURE 1:**
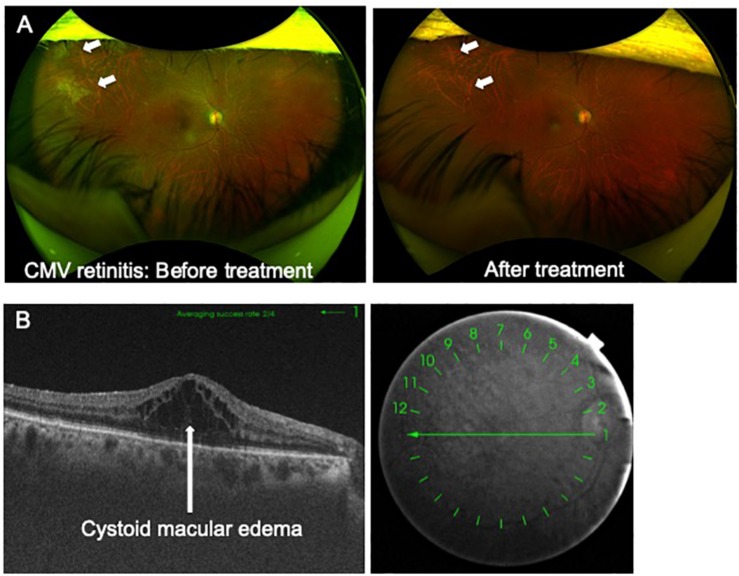
**(A)** Advances in imaging technology. Cytomegalovirus (CMV) retinitis in a patient infected by HTLV-1. Widefield fundus cameras can reveal the lesion (CMV retinitis, white arrow) at the peripheral retina and can track the lesion during follow-up. **(B)** Advances in imaging technology. Optical coherence tomography clearly captures cystoid macular edema accompanied by HTLV-1 uveitis.

Imaging of ocular lesions is important to identify HTLV-1-associated ocular lesions. HU results in retinal vasculitis, while ATL induces intraocular infiltration and cytomegalovirus (CMV) retinitis at the periphery of the retina. For instance, careful attention should be directed toward possible necrotic lesions of CMV retinitis arising at the peripheral retina. To follow-up such CMV lesions in the periphery of the retina, widefield fundus images are indispensable (Figure 1A).

Optical coherence tomography (OCT) is one of the most important modalities applied in ophthalmology. OCT is a non-invasive diagnostic technique that enables visualization of the retina in a cross-sectional view. OCT has greatly evolved the practice of ophthalmology, as cross-sectional mapping of the retina has provided large amounts of information and new insights.

OCT is useful for the diagnosis of HTLV-1-associated ocular lesions such as HU and ATL. For example, HU is sometimes accompanied by cystoid macular edema (CME), which significantly affects visual acuity. OCT can clearly reveal the features of CME (Figure 1B). As for ATL-related ocular manifestations, we first described the details of ATL infiltration captured with OCT, which identified ATL cell infiltrates below the retinal pigment epithelium, resulting in multiple, knob-like infiltrations (Kamoi and Ohno-Matsui, 2020). ATL patients often suffer from opportunistic infections such as CMV retinitis. CMV destroys the retinal structure and OCT can capture the details.

### Advances in Diagnostic Technology

Marked advances in molecular technologies have been made in the last decade. The diagnostic methods of uveitis in clinical practice have been significantly changed by the application of molecular biological technologies, particularly polymerase chain reaction (PCR).

PCR allows accurate analysis of small volumes of ocular samples (such as aqueous humor or vitreous fluid). However, several applications of the technique have been necessary to check for the various causative pathogens. Recently, our group developed multiplex and broad-range PCR methods (Sugita et al., 2008, 2011, 2012, 2013). These techniques have impacted the differential diagnosis of uveitis, allowing prompt and accurate analysis of multiple pathogens (herpes simplex virus-1 and -2, varicella-zoster virus, Epstein-Barr virus, CMV, human herpes virus 6-8, HTLV-1, toxoplasma, tuberculosis, syphilis, bacteria, and fungi) in a single test using small-volume ocular samples (Mochizuki et al., 2017). Diagnosis by this new PCR method facilitates prompt and appropriate therapy. The usefulness of this method was confirmed in multi-center studies (Sugita et al., 2013; Nakano et al., 2019) and the importance of the method has been introduced to ophthalmologists (Dabil et al., 2001; Minkus et al., 2019).

As for the diagnosis of HTLV-1-associated ocular lesions, especially among ATL patients, our PCR method is quite useful. ATL-related ocular manifestations are very complicated, because the ocular presentations of infection and infiltration are sometimes difficult to distinguish. Identifying or excluding pathogenic agents is a very important clue for the diagnosis of ATL ocular manifestations. If pathogenic agents can be identified, pathogen-specific drugs (antibiotics, antifungal agents, and antiviral agents) can be given promptly, leading to protection against ocular tissue damage by pathogens. If pathogenic agents fail to be identified, ATL infiltration should be considered. As ocular infiltration of ATL cells is rapid and results in severe destruction of intraocular tissues, addressing this with adequate treatment is critical to prevent vision loss.

### Advances in Drug Delivery

Delivery of pharmacotherapies by intravitreal injection (IVI) represented a turning point in the treatment of various of ocular conditions, including age-related macular degeneration, diabetic macular edema, proliferative diabetic retinopathy, retinal vein occlusion, pathological myopia, and uveitis. In the early 1900s, IVI was introduced for procedures addressing retinal detachment. In recent years, the use of IVI has grown exponentially, due to the progressive expansion of clinical applications.

ATL patients often have multiple organ disorders such as kidney failure due to the disease itself or side effects of drugs, and systemic treatment should be avoided in such cases. In this situation, IVI of the drug offers a useful delivery method to lesions in the eye, while reducing overall burden on the body. Rapid progression of a lesion should stop with immediate treatment and the most effective approach for this situation is delivering the drug through IVI, as pharmaceutical agents are able to reach the lesion directly. Progressive ocular lesions such as ocular infiltration of ATL easily leads to vision loss and thus countermeasures should be enacted immediately (Kamoi and Ohno-Matsui, 2020). Delivery of drugs through IVI is thus still developing and represents a promising method.

As for relatively new delivery methods, sub-Tenon injection has been noted. This delivery method is mainly applied for HU and the accompanying CME. In cases of severe vitreous opacity and CME in HU, in which eye drops or oral steroid administration are not sufficient, injection of steroid such as triamcinolone acetonide into the sub-Tenon space appears much more effective.

## HTLV-1 Uveitis and Relevant Manifestations

### HTLV-1 Uveitis

**FIGURE 2:**
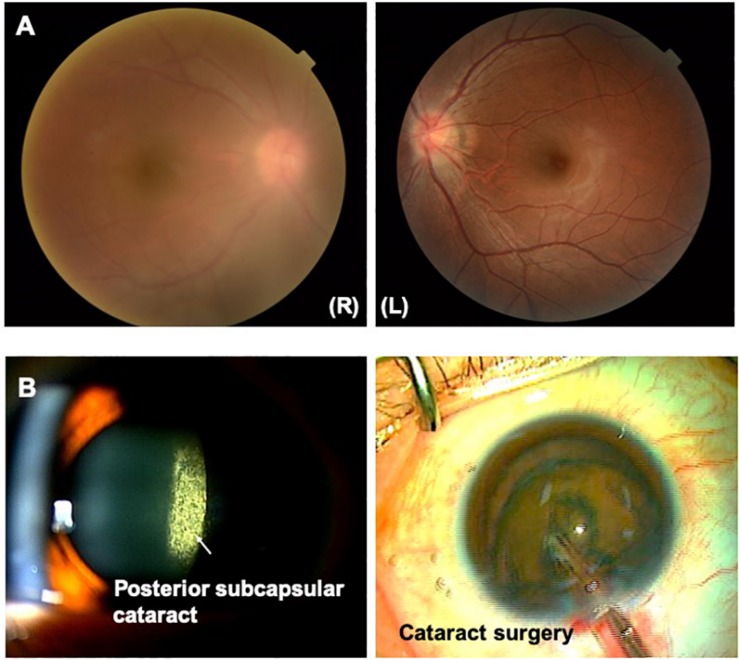
**(A)** HTLV-1 uveitis (HU). Vitreous opacity is seen in the right eye. Vitreous opacity disturbs light penetration, preventing clear and detailed visualization of the retina. **(B)** Ocular complications secondary to HU. Posterior subcapsular cataract secondary to HU is clearly evident. A phaco dislocation technique is used to perform cataract surgery with a low level of aggression and high degree of safety.

HTLV-1 uveitis (HU) was the first ocular disease directly associated with HTLV-1 infection from the perspective of clinical and basic research in the early 1990s. According to the anatomical classification of intraocular inflammation, most cases of HU are classified as intermediate uveitis, followed by posterior uveitis. The main clinical feature of HU is the moderate to high infiltration of inflammatory cells into the vitreous, resulting in vitreous opacities [Kamoi and Mochizuki, 2012a, b, 2016, 2017; Figure 2A(R)]. As a result, smooth light penetration is disturbed, leading to decreased vision and making patients aware of foggy vision and floaters. As for other clinical features, anterior uveitis and retinal vasculitis are also seen, but the inflammatory grades are mild.

The peak age at uveitis onset was in the 50s followed by the 60s for women, and in the 60s followed by the 70s for men, according to one recent survey (Terada et al., 2017b), which showed that age at onset had increased compared with previous research. Women greatly outnumbered men among HU patients (Takahashi et al., 2000; Terada et al., 2017a). Recurrence of uveitis was seen in 30–50% of HU patients (Nakao and Ohba, 1993; Takahashi et al., 2000; Terada et al., 2017a).

As for ocular complications secondary to HU, cataracts represent the most common ocular complication, followed by glaucoma, CME, and epiretinal membrane (Terada et al., 2017a). In terms of systemic complications, an association between HU and Graves’ disease has been reported continuously (Nakao et al., 1994; Yamaguchi et al., 1994; Miyanaga et al., 2009; Terada et al., 2017a; Nakao et al., 2018). An association between HU and HAM has also been pointed out, as mentioned in the previous section. On the other hand, several reports have indicated that the association between HU and ATL is less relevant (Terada et al., 2017a; Nakao et al., 2018).

A series of studies has been conducted on the pathogenesis of HU. According to analyses of intraocular fluid, HTLV-1-infected cells infiltrate the eye, where HTLV-1 provirus (Mochizuki et al., 1992a; Ono et al., 1997) and virus particles associated with the cells (Sagawa et al., 1995) have been detected. The T-cell receptor of intraocularly infiltrating HTLV-1-infected T cells was identified as polyclonal (Masuoka et al., 1995). In the peripheral blood, viral load among HU patients is significantly higher than that among asymptomatic carriers (Ono et al., 1995). The proviral load in ocular fluid is higher than that in peripheral blood (Ono et al., 1997). HTLV-1-infected T cells that have infiltrated into the eye produce large amounts of various inflammatory cytokines and the production of cytokines is able to be suppressed by steroids (Sagawa et al., 1996).

According to basic research, HTLV-1 has the potential to infect human ocular cells (Liu et al., 2006; Uchida et al., 2019). These investigations have revealed that HTLV-1-infected retinal pigment epithelial (RPE) cells disturb homeostasis in the retina. HTLV-1 infection correlated with elevated expression of intercellular adhesion molecule (ICAM)-1 on the surface of RPE, which may lead to the tropism of RPE for HTLV-1 (Liu et al., 2006).

As for animal models, the first animal model of HU was established in the early 1990s. HTLV-1-infected rabbits showed uveitis after a latency of 3.5 years. The ophthalmic features of rabbits were quite similar to HU and HTLV-1-associated ocular conditions of humans from the perspectives of corneal opacity, anterior inflammation, vitreous opacity, and retinal destruction (Fukushima et al., 1993; Taguchi et al., 1993). Using an HTLV-1-infected mouse model, the involvement of autoimmune mechanisms in the induction and prolongation of HU was suggested by showing that epitopes of HTLV-I antigens were cross-reactive with epitopes of retinal antigens (Fukushima et al., 1994).

Based on the above series of basic studies, the current explanation of the mechanisms underlying HU is as follows. In HU patients, the number of HTLV-1-infected cells increases in the peripheral blood and infected cells significantly accumulate in the eye. Accumulated HTLV-1-infected cells in the eye produce various inflammatory cytokines and chemokines, leading to damage to ocular tissues. From another perspective, HTLV-1-infected RPE cells contribute to the breakdown of immune homeostasis in the eye. Cross-reactivity between HTLV-1 and retinal antigens would also contribute to HU. However, what causes the increase in infected T cells in peripheral blood and why accumulation occurs in the eye both remain unclear. Further investigation is needed to identify the mechanisms of HU in detail.

In the clinic, HU is diagnosed by the presence of uveitis, confirmation of HTLV-1 infection (detection of positive serum HTLV-1 antibody by an enzyme antibody method or particle agglutination method, detection of HTLV-1 protein by western blot, or detection of a provirus pX region by PCR), and exclusion of other etiologies of defined uveitis (Kamoi and Mochizuki, 2012b, 2016, 2017). For instance, sarcoidosis, the leading cause of uveitis in Japan, shows ocular symptoms similar to HU (especially vitreous opacities), and is therefore particularly important as an exclusion diagnosis in Japan. To exclude other etiologies of uveitis, serum angiotensin-converting enzyme, interferon-gamma release assay such as quantiferon, and chest X-ray/CT examination must be performed for diagnosis.

HU is considered to be caused by inflammatory cytokines produced from HTLV-1-infected cells, which significantly accumulate in the eyes of the patient. Topical and/or oral corticosteroid treatment is therefore effective to treat HU patients, suppressing cytokine production by the HTLV-1-infected T cells causing intraocular inflammation (Sagawa et al., 1996; Kamoi and Mochizuki, 2012a, b, 2016, 2017). Clinical management according to the degree of ocular inflammation should be performed. HU with mild ocular inflammation can be managed by topical non-steroidal agents or corticosteroids. Sub-Tenon injection of steroids is chosen when the patient displays moderately inflammatory activity in the vitreous cavity or CME formation. If vitreous inflammatory activity and retinal vasculitis are severe, oral steroids are given, but long-term administration of systemic steroid should be avoided.

As for ocular complication secondary to HU, advanced cataracts require cataract surgery. Cataract surgery in uveitis must be performed with a low level of aggression and a high level of safety (Kamoi and Mochizuki, 2008) by selecting adequate techniques (Kamoi and Mochizuki, 2008, 2010; Jacob, 2019; Figure 2B). As glaucoma is also a common ocular complication secondary to HU, non-invasive treatments such as topical eye drops are mainly selected as the first line to reduce ocular pressure. However, topical eye drops are insufficient, and invasive treatments such as trabeculotomy or trabeculectomy should be consider (Kesav et al., 2019).

### Keratoconjunctivitis Sicca

Keratoconjunctivitis sicca (KCS), also known as dry eye syndrome, involves chronic, bilateral discomfort of the conjunctiva and cornea due to inadequate lubrication and a reduced tear meniscus with a tear breakup time under 10 s. KSC is known to be associated with systemic diseases such as Sjögren’s syndrome, rheumatoid arthritis (RA) and lupus.

**FIGURE 3:**
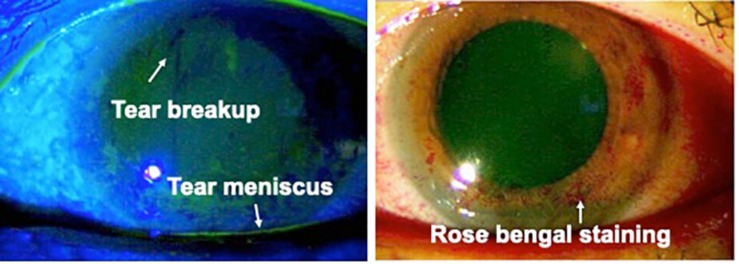
Keratoconjunctivitis sicca (KCS) in an HTLV-1-infected patient. Reduction of the tear meniscus is seen (white arrow). Tear breakup time in this patient was 2 s. Rose bengal staining shows damage to the ocular surface (reproduced with permission from Ide et al., 2016).

In the late 1980s, basic research in animal models showed tear film alteration with Sjögren’s syndrome in HTLV-1-related transgenic mice (Green et al., 1989). From the 1990s, an association between Sjögren’s syndrome and HTLV-1 started to be reported in clinical studies (Eguchi et al., 1992; Terada et al., 1994). The prevalence of decreased tear breakup time among HTLV-1 carriers was significantly higher than that among non-carriers (Yamamoto et al., 1999). The prevalence of KCS among HTLV-1 carriers was found to be around 30% according to research from Martinique and Brazil (Pinheiro et al., 2006; Rathsam-Pinheiro et al., 2009, 2019). HTLV-1 proviral loads are higher in patients with KCS and may represent a relevant biological marker of disease (Castelo Branco et al., 2001). Patients with HAM manifest significantly higher frequencies of KCS than asymptomatic HTLV-1-infected individuals, along with immunologic alterations. Levels of activated CD8-positive T cells could be used as a prognostic marker of inflammatory disease manifestation to follow-up HTLV-1 carriers (Pinheiro et al., 2006). Considering such reports regarding clinical biological markers of KCS among HTLV-1-infected individuals, the mechanisms of KCS in HTLV-1 carriers might differ from the typical ocular mechanism in Sjögren’s syndrome, because the immunological alterations are not caused by autoimmunity, but rather by HTLV-1 infection. Typical findings for KCS under HTLV-1 infection are shown in Figure 3.

As for treatment, the definitive treatment for KCS in HTLV-1 carriers has not been established. The current approach is symptomatic therapy. Lubrication of the ocular surface is needed to avoid the development of superficial punctate keratitis and corneal ulcers.

### Interstitial Keratitis

Chronic interstitial keratitis (IK) related to HTLV-1 infection was first reported in the early 2000s (Buggage et al., 2001; Merle et al., 2001). Keratitis was reported in 10% of HTLV-1-infected patients and was strongly associated with HAM (Merle et al., 2001). Patients with keratitis show no subjective symptoms, such as pain, dryness of the eyes, or vision loss, but round or patchy whitish opacities confined bilaterally to the anterior stroma are observed in the cornea. Other corneal lesions are also seen at the periphery of the cornea and spare the visual axis, without ulceration or neovascularization.

The mechanism underlying these corneal lesions could be linked to lymphoplasmacytic infiltration of the stroma leading to corneal opacities (Merle et al., 2001). Corneal lesions have also been suspected to be due to hypergammaglobulinemia induced by HTLV-1 infection (Patronas et al., 2006). Topical administration of steroids thus does not achieve response from the corneal lesions, which remain chronic.

### Optic Neuritis

Optic neuritis was first reported accompanying HAM (Yoshida et al., 1998). HTLV-1-associated optic neuritis is diagnosed after excluding all other diseases that cause optic neuritis, such as multiple sclerosis (MS), neuromyelitis optica (NMO), infection and tumor infiltration. Laboratory data and radiological features thus need to be investigated in detail. An oligoclonal immunoglobulin G band in cerebrospinal fluid and brain lesions on MRI are detectable in both HAM and MS, making MS difficult to differentiate from HAM, especially in HTLV-1 endemic areas. Anti-AQP4 antibody in serum was recently identified as a specific laboratory marker for NMO (Wingerchuk et al., 2007), and clinical and basic research has indicated that NMO is a disease entity distinct from MS. Laboratory checks for anti-AQP4 antibody are thus needed in diagnosis and treatment to determine whether interferon should be applied as a therapy, since the administration of interferon is considered effective for HAM but not for NMO (Araujo and Silva, 2006; Koga et al., 2009).

Recurrent NMO associated with HTLV-1 infection has been reported (Olindo et al., 2010), and basic research has shown that latent HTLV-1 infection could lead to TAX1BP1 antigen presentation and the production of anti-AQP4 antibodies through T-cell-mediated mechanisms (Kampylafka et al., 2015). On the other hand, reports have shown that AQP4 antibody is not associated with the pathogenesis of HAM/TSP, and HTLV-1 infection is not associated with the development of AQP4-Ab (von Glehn et al., 2012). Further research is needed to elucidate the association between HTLV-1 infection and optic neuritis.

### Overlap Syndrome

Overlap syndrome refers to HTLV-1-infected patients suffering more than one HTLV-1-associated inflammatory condition. HTLV-1 infection is considered to affect systemic immunological homeostasis, because T-cells are activated by HTLV-1 infection and cytokines and chemokines are produced from HTLV-1-infected cells. These alterations result in damage to resident cells in human. Clinically, overlap of HU and HAM is seen in less than 15% of cases (Nakao et al., 2018). KCS and IK, which are partially related to inflammation, are more frequent among HAM patients (Merle et al., 2001; Pinheiro et al., 2006; Castro-Lima Vargens et al., 2011).

According to basic investigations, transgenic mice expressing HTLV-1 Tax develop inflammatory arthropathy, and transgenic rats expressing HTLV-1 env-pX develop Sjögren’s syndrome, arthropathy, vasculitis, and polymyositis (Nakamaru et al., 2001). Regulatory T cells in HTLV-1 bZIP factor transgenic mice are functionally impaired, resulting in systemic inflammation (Satou et al., 2011). A wide variety of factors seem to contribute to this overlap syndrome.

## ATL-RELATED OCULAR MANIFESTATIONS

Since ATL-related ocular manifestations are rare and much less frequent than the prevalence of HU, manifestations have been documented in sporadic case reports or case series. Although several ocular manifestations have been identified, the frequencies remain unclear. Our recent nationwide survey in Japan revealed the frequency of ATL-related ocular manifestations against the background of the highest endemic rate among developed countries. According to the survey, the most frequent manifestation is intraocular infiltration, followed by opportunistic infection (with all cases involving CMV retinitis), KCS, and scleritis (Table 1).

**TABLE 1:** ATL-related ocular manifestations.

ATL-related ocular manifestations	*n* = 48	%
Intraocular infiltration	22	45.8
Opportunistic infection	19	39.6
(Cytomegalovirus)	(19)	(100.0)
(Herpesvirus)	(2)	(10.5)
(Toxoplasma)	(1)	(5.3)
Dry eye	3	6.3
Scleritis	2	4.2
Uveitis	1	2.1
Anemic retinopathy	1	2.1

**Our nationwide survey (Kamoi et al., 2018) identified intraocular infiltration and opportunistic infections as common ATL-related ocular lesions. All cases of opportunistic infection involved cytomegalovirus retinitis.**

### Infiltration

Compared with other forms of leukemia, ATL cells are much more adept at infiltrating the eyes (Kamoi et al., 2018). Previous case reports have indicated that ATL cells have the potential to infiltrate into various tissues in the eye such as the orbit (Mori et al., 2003; Fares et al., 2018), conjunctiva (Takahashi et al., 1993), lacrimal glands (Sabundayo et al., 2018), cornea (Buggage et al., 2001; Miyamura et al., 2009), vitreous humor (Ohba et al., 1989; Kohno et al., 1993; Hirata et al., 2002; Maruyama et al., 2013), uvea (Mori et al., 2003), retina (Ohba et al., 1989; Kohno et al., 1993; Hirata et al., 2002), choroid (Liu et al., 2010), and optic nerve (Yamamoto et al., 1994).

**FIGURE 4:**
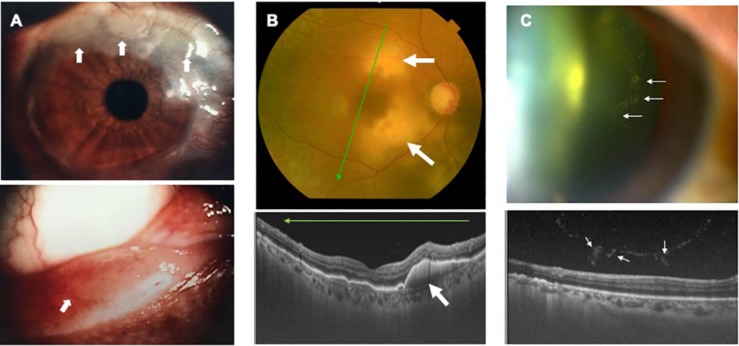
Knob-like ATL cell multiple ocular infiltration (KAMOI) sign. KAMOI sign (**A,B**; white arrows) can be seen at the bulbar conjunctiva around the corneal limbus and at the palpebral conjunctiva around the lacrimal punctum **(A)** (reproduced with permission from Kamoi et al., 2016) and retina **(B)**. Infiltrated ATL cells in the vitreous tend to form multiple clusters **(C)**.

Infiltration of ATL cells into the eye provides a specific sign, which we have termed the “knob-like ATL cell multiple ocular infiltration” (KAMOI) sign. At the ocular surface, this KAMOI sign can be seen most prominently at the bulbar conjunctiva around the corneal limbus and at the palpebral conjunctiva around each lacrimal punctum (Kamoi et al., 2016; Mochizuki et al., 2017; Figure 4A). KAMOI sign can also be detected in the retina (Kamoi and Ohno-Matsui, 2020; Figure 4B). Taken together with other clinical reports (Takahashi et al., 1993; Mori et al., 2003; Yoshikawa et al., 2005), KAMOI sign is one of the most characteristic forms of ATL infiltration into the eye. When considering the mechanisms underlying this characteristic infiltration, analysis of the vitreous humor appears informative. Slit-lamp and OCT examinations show that infiltrating ATL cells in the vitreous tend to form multiple clusters (Figure 4C). The mechanisms behind this cluster formation remain unclear, but might involve the high expression of ICAM-1 on ocular cells and ATL cells (Liu et al., 2006).

As for methods of imaging diagnosis, the slit-lamp scope and fundus scope have been useful to detect infiltrating lesions in the eye. OCT, one of the most recently developed modalities, is now able to identify ATL infiltration in detail (Figure 4B). Recent findings show that ATL cells can penetrate Bruch’s membrane, and accumulate under the RPE layer and form the KAMOI sign during intraocular infiltration (Kamoi and Ohno-Matsui, 2020; Figure 4B).

As for laboratory diagnosis, methods for the analysis of ocular samples have evolved. Although cytology offers a precise method for detecting ATL cells, clinically obtainable ocular samples are limited in terms of quantity. PCR methods thus offer clinical advantages in ophthalmologic diagnosis (Mochizuki et al., 2017). Detection of HTLV-1 proviral DNA and monoclonal T-cell receptor gamma chain gene rearrangements by PCR provide key clues to confirming ATL infiltration (Kamoi et al., 2016). In parallel, exclusion of infection is needed from the perspective of practical therapy, as the choice of drugs differs completely among infiltration, infection and inflammation. Multiplex and broad-range PCR should thus be performed to exclude non-contributory pathogens.

Therapy for intraocular infiltration previously relied on systemic ATL therapy such as intensive multi-agent chemotherapy (Tsukasaki et al., 2012), interferon-a combined with zidovudine (Gill et al., 1995), anti-CCR4 antibody (mogamulizumab) (Ishida et al., 2012), hematopoietic stem cell transplantation (Zell et al., 2016), and Tax peptide-pulsed dendritic cell vaccine (Suehiro et al., 2015). These advanced therapies are considered effective for ATL ocular infiltration. However, severe and rapid sight-threatening infiltration into key ocular tissues such as the retina can lead to critical and irreversible vision loss. Immediate topical treatment is therefore imperative to preserve vision. As for topical therapies for intraocular infiltration, focal radiation has been reported as effective (Yamamoto et al., 1994). We have now performed IVI of methotrexate in combination with focal radiation (Kamoi and Ohno-Matsui, 2020), and confirmed this combination topical therapy as effective for ATL ocular infiltration (Kamoi and Ohno-Matsui, 2020).

### Infection

According to our nationwide survey, the most frequent infectious manifestation among ATL patients was CMV retinitis (Kamoi et al., 2018; Table 1). CMV is well established as the most frequent pathogen underlying opportunistic infections among ATL patients (Suzumiya et al., 1993; Maeda et al., 2015), and CMV infection occurs more frequently in patients with ATL than in those with other leukemias (Funai et al., 1995). Among ATL patients, HTLV-1-infected CD4-positive T cells can transform into malignant cells, losing the normal function of CD4-positive T cells. As a result, cellular immunity is severely impaired, resulting in frequent CMV infection.

**FIGURE 5:**
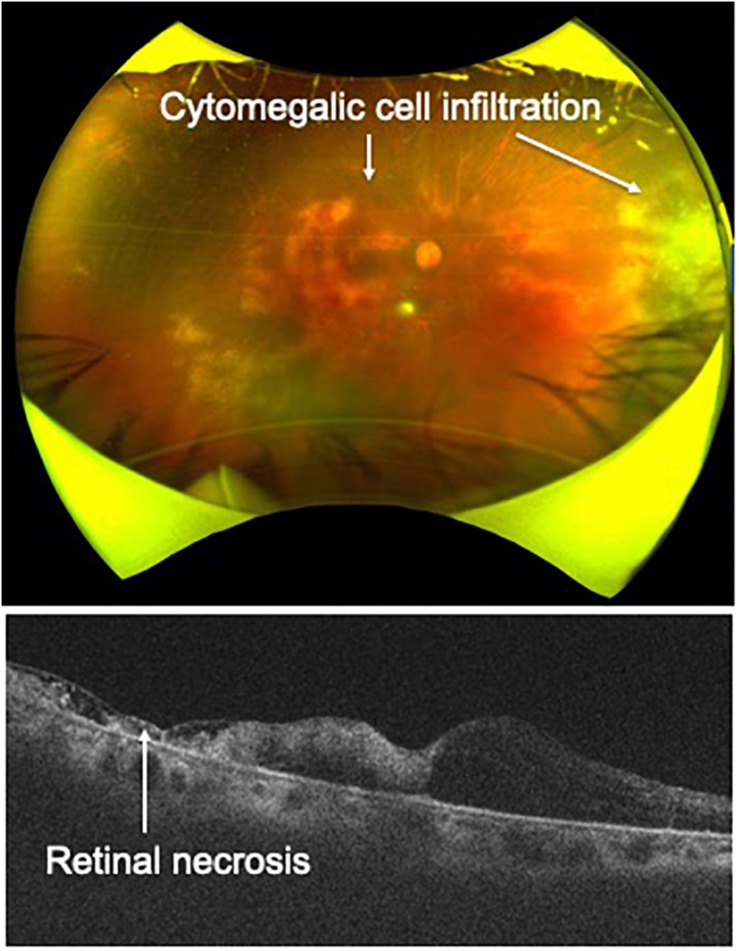
Cytomegalovirus retinitis. Cytomegalic cell infiltration and accompanying retinal necrosis are captured by widefield fundus camera and OCT in a patient with ATL.

As for imaging diagnosis, widefield fundus photography and OCT are helpful for identifying CMV retinitis, as these techniques can capture cytomegalic cell infiltration and accompanying retinal necrosis (Figure 5). Detection of granular yellow-white lesions and retinal destruction are clues to the clinical diagnosis. To confirm CMV retinitis from laboratory tests, obtaining ocular samples and performing multiplex and broad-range PCR are needed, as ATL patients have an immunocompromised condition and other types of opportunistic infection (such as fungal infection) should be excluded. In terms of treatment, systemic administration of antiviral agents such as ganciclovir and valganciclovir is effective. In severe and rapidly progressive cases, IVI of ganciclovir is effective to prevent lesion progression.

Our nationwide survey identified co-infections with CMV such as herpes simplex virus and toxoplasma can be seen in some ATL patients (Table 1). In addition, other opportunistic infections such as cryptococcal choroiditis have been reported as ATL-related ocular manifestations (Yamada et al., 2019). Therefore, performing multiplex and broad-range PCR of ocular samples can provide crucial information relevant to the selection of pharmacotherapies.

### Inflammation (Uveitis)

Simultaneous development of ATL and ocular inflammation has been reported (Hirano et al., 2017; Nakao et al., 2018). Invasion of inflammatory cells into the eye results in breakdown of the blood ocular barrier (BOB). Searching for biomarkers of simultaneous occurrence, breakdown of the BOB correlated with increased ATL cells in peripheral blood. This means that rapid expansion of ATL cells is one reason for breakdown of the BOB (Hirano et al., 2017). This type of ocular inflammation is called ATL cell-induced uveitis (AIU) (Hirano et al., 2017).

In AIU, ophthalmic examinations can detect infiltrating cells in the anterior chamber/vitreous, and retinal vasculitis. Diagnosis requires exclusion of ATL infiltration and opportunistic infections. Examination of ocular samples using PCR methods and cytology is useful. As for treatment, AIU often responds to topical steroid eye drops, but if the eye drops do not achieve adequate response, systemic steroids should be considered.

### Scleritis

**FIGURE 6:**
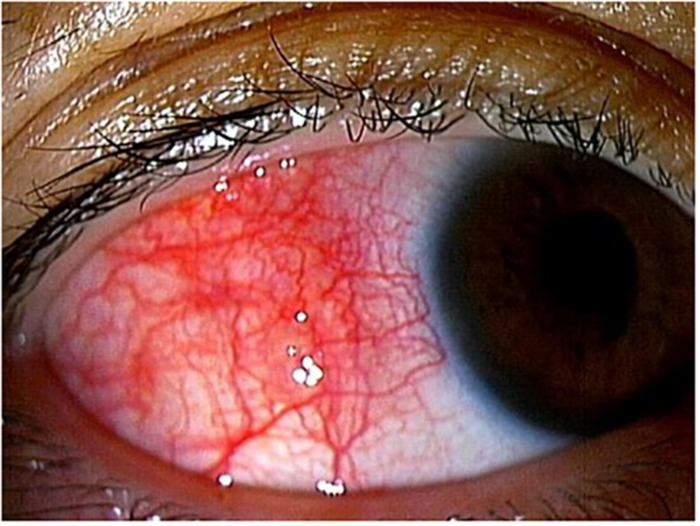
ATL-related scleritis. Conjunctival and scleral injections are evident.

ATL-related scleritis (Figure 6) has been reported by several groups (Goto et al., 1993; Oono et al., 2009; Larson et al., 2012). The mechanisms remain unclear, but topical steroids are effective for temporarily reducing scleritis (Oono et al., 2009). This suggests that immune-mediated reactions may play a role in the mechanisms of ATL-related scleritis.

For treatment, daclizumab (a monoclonal antibody directed against a chain of interleukin-2 receptor (IL-2R) and denileukin diftitox (an immunotoxin fusion protein that targets IL-2R) reportedly improve scleritis in patients with underlying ATL (Larson et al., 2012). This suggests that the ATL cell itself might contribute to the mechanisms underlying scleritis, but further investigation is needed.

## Current Concerns

Drug-induced uveitis is a well-known phenomenon among ophthalmologists. Recently, a variety of drugs have been applied to HTLV-1 carriers, but the adverse effects of these drugs on the eye have not been fully characterized. Although HTLV-1 carriers face a credible threat of intraocular inflammation, safety assessments of drugs have not been completed and current guidelines do not mention whether patients should be checked for HTLV-1 infection prior to the administration of drugs.

Nowadays, treatment with molecularly targeted drugs such as biologics has been introduced in consideration of the mechanisms underlying the inflammatory diseases. Tumor necrosis factor (TNF)-blocking biologics were first introduced for inflammatory diseases, with other biologics such as IL-6-blocking agents introduced subsequently. Recently, we experienced a case in which a patient with RA and HTLV-1 infection received an IL-6-blocking agent, which reactivated HTLV-1 and induced HU in the eye (Terada et al., 2017b). We have since been cautious of the risk of newly developed molecularly targeted drugs reactivating HTLV-1 and inducing ocular inflammation. Therefore, we have first tried to clarify the safety of infliximab as the first biologic drug introduced clinically. Our results have revealed that infliximab does not exacerbate HTLV-1-associated conditions such as inflammation in the eye *in vitro* (Uchida et al., 2019). Further assessment of other drugs is needed to avoid induction of HTLV-1-associated ocular diseases, and to maintain vision in HTLV-1 carriers.

## Unmet Research Needs

Although great advances in ophthalmology have been seen since the discovery of associations between HTLV-1 and ocular diseases, various issues remain to be resolved. In terms of understanding the mechanisms involved, basic research into HTLV-1-associated diseases is still nowhere near complete, with remaining issues including how HTLV-1-infected cells break the BOB, and what roles HTLV-1-infected cells play in the eye. Although multiplex and broad-range PCR have been applied to HTLV-1-associated ocular diseases and the accuracy of diagnosis has vastly improved, diagnosis remains a complicated process requiring diagnosis by exclusion. In the future, advancing technologies such as flow cytometric analysis using ocular fluid (Akaike et al., 2016) might prove fruitful for identifying pathogenic infected cells more specifically. Biomarkers allowing direct diagnosis is clearly a desirable goal of future research. As for treatment, current options for HU are overly dependent on steroids, and side effects such as secondary cataracts and secondary glaucoma thus remain problematic. In the future, HTLV-1-infected cell-targeted drugs should be developed to treat HU much more safely and efficiently. ATL cell-targeted drugs should also be developed to address ATL infiltration.

## Conclusion

Looking back on the history of HTLV-1-associated ocular diseases, information accumulated from all over the world has helped to advance medical care for HTLV-1-associated ocular diseases since the discovery of the relationship between HTLV-1 and ocular diseases. However, HTLV-1-associated ocular diseases have been mainly reported from limited areas, and numbers of patients with HTLV-1-associated ocular diseases remain unclear for areas such as Africa and Australia. This may be due to low awareness among ophthalmologists in these areas regarding HTLV-1-associated ocular disease. In addition, HTLV-1-associated ocular diseases have now spread globally in conjunction with the migration of HTLV-1-infected individuals. An overview of these diseases in vast areas of the world is therefore needed, to provide information on HTLV-1-associated ocular diseases and prevent visual loss around the globe.

As shown in this article, continued accumulation of knowledge from recent clinical and basic research has brought many new insights. Application of advances in ophthalmic technologies has brought about much more sophisticated medical care. Recent diagnostic technologies such as multiplex/broad-range PCR and OCT and treatment methods such as the direct approach through IVI of the drug have contributed to improvements in the diagnosis and treatment of HTLV-1-associated ocular diseases. In the future, development of techniques such as flow cytometry might represent the next step in advancing diagnostic methods, but the fact that only small-volume samples are able to be obtained from the eye must be addressed. In terms of treatment methods, development of new specific topical drugs based on the pathogenesis of HTLV-1-associated ocular diseases might be desirable, allowing direct injection into the eye. To provide forward-looking medical care for HTLV-1 in ophthalmology, various avenues of research must be explored to improve the quality and maintenance of vision among HTLV-1-infected patients. The constant effort of ophthalmologists is needed to achieve excellent vision for HTLV-1-infected patients.

## Author Contributions

KK conceptualized and wrote the manuscript.

## Conflict of Interest

The author declares that the research was conducted in the absence of any commercial or financial relationships that could be construed as a potential conflict of interest.
